# Supplementary material for: Body-Weight Fluctuations and the Association Between the Consumption of Protein-Rich Foods and the Incidence of Metabolic Syndrome Among Middle-Aged Women in Korea
Source: Healthcare (Basel). 2025 Mar 24;13(7):709. doi: 10.3390/healthcare13070709 (PMC11988784; doi:10.3390/healthcare13070709)
Supplement: Supplementary file 1 [file healthcare-13-00709-s001.zip › healthcare-3476689-supplementary.pdf]

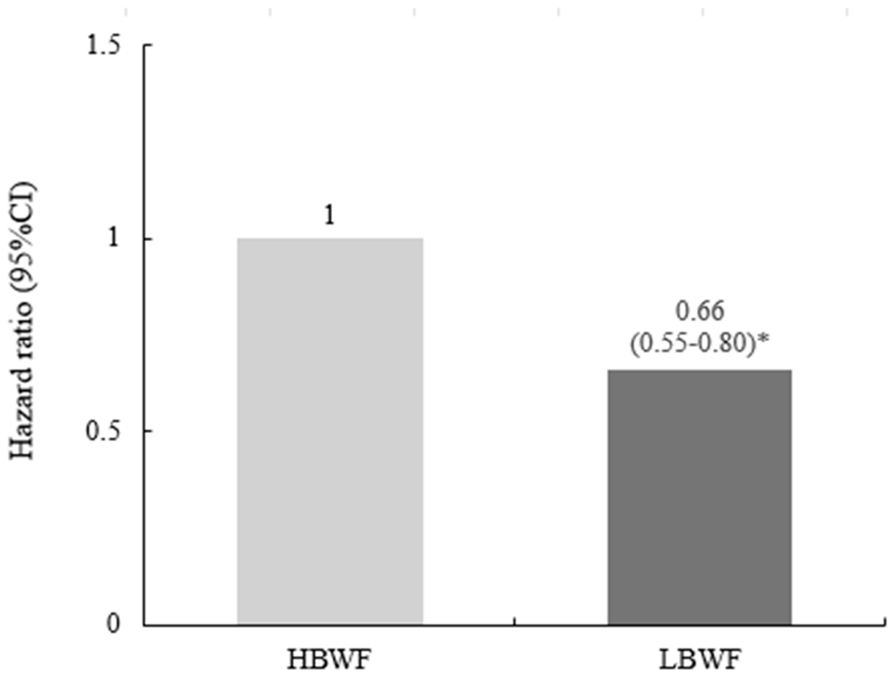

Supplementary Figure 1. Incidence of metabolic syndrome by body weight fluctuations among premenopausal subjects. The hazard ratio (95% confidence interval) and p value of the LBWF group were calculated using the HBWF group as the reference. *p value <0.001. LBWF, low body weight fluctuation; HBWF, high body weight fluctuation.
